# Paying Attention to Women's Ageing Bodies in Recovery From Substance Use

**DOI:** 10.3389/fpsyt.2022.890784

**Published:** 2022-05-17

**Authors:** April Shaw, Gerda Reith, Lucy Pickering

**Affiliations:** ^1^National Addiction Centre, Institute of Psychiatry, Psychology and Neuroscience, King's College London, London, United Kingdom; ^2^School of Social and Political Sciences, College of Social Sciences, University of Glasgow, Glasgow, United Kingdom

**Keywords:** mid-life and older women, substance use, ageing, bodies, recovery, health, menopause

## Abstract

**Background:**

Health-related research on women who use drugs (WWUD) tends to focus on reproductive and sexual health and treatment. Missing from the picture is an exploration of mid-life and older women's bodily experiences of transitioning from long-term substance use into recovery. While there are a growing number of studies that explore the intersection of drug use and ageing, the gaps in analysis lie in the intersections between drug use, recovery, ageing, gender, and the body.

**Methods:**

In-depth qualitative interviews were undertaken with 19 women in the UK who self-identified as “in recovery” from illicit drug use. The interviews were transcribed verbatim and analysed using Braun and Clarke's thematic analysis techniques. The study received ethical approval from the University of Glasgow.

**Results:**

Key findings from the interviews relate to the women's personal sense of power in relation to current and future health status, the challenges they endured in terms of ageing in recovery and transitioning through the reproductive life cycle, and the somatic effects of trauma on women's recovery. The findings demonstrate that health in recovery involves more than abstinence from drugs.

**Discussion:**

Moving from the body in active drug use to the body in recovery is not without its challenges for mid-life and older women. New sensations and feelings—physical and mental—must be re-interpreted in light of their ageing and drug-free bodies. This study reveals some of the substantive sex-based differences that older women in active drug use and recovery experience. This has important implications for healthcare and treatment for women in drug services and women with histories of drug use more generally.

## Introduction

An androcentric approach to health has been prevalent within medical research and this is particularly true of substance use research (1). A particularly neglected research area in the field of substance use relates to the bodily and embodied experiences of mid-life and older women in recovery from illicit drug use. This paper's aim is to pay attention to the voices of women ageing into recovery and provide a deeper understanding of their experiences of drug-free and ageing bodies. This paper specifically focuses on women's experiences of *bodily recovery* from unmanageable substance use.

As the body ages its ability to process drugs changes (2). Age-related changes in physiology and long-term drug use can increase adverse drug reactions in older people, exacerbate a decline in organ function, and increase respiratory disorders and cancers (3). Higher rates of blood-borne viruses (BBVs), and physical and mental health morbidity are reported among older people who use drugs compared to their younger cohorts (4). In terms of gender differences, older women who use drugs exhibit poorer overall health status and more chronic physical and mental health problems than older men who use drugs (5). Women report higher rates of heart disease, circulation problems, asthma, bladder problems, colitis/bowel problems and arthritis than their male counterparts (6). Women who use opioids and alcohol report higher levels of psychological distress, including anxiety, depression and panic disorder than men (5, 6). Adverse childhood experiences are associated with lifetime mental health conditions and alcohol and substance use among older people (7, 8) and women who use heroin are potentially more prone to mental health conditions than their male counterparts given that many are likely to have experienced emotional, physical and sexual violence and exploitation (9, 10). While existing research suggests gendered differences in physical and mental health problems among older people who use substances, the lack of focus on gender differences is a limitation in understanding the complex physical and mental health needs of older men and women who use opiates, alcohol and other drugs (5, 11).

For most women, the reproductive lifecycle is one of the biological conditions that distinguishes their bodily experiences from men. Women who use drugs can experience amenorrhoea (the absence or cessation of menses in women of reproductive age) (12). Perceived infertility due to drug-induced amenorrhoea may result in unplanned pregnancy (12, 13) during active drug use and following abstinence when the women's menstrual cycle begins again. Though limited, evidence suggests that older women who use drugs may be at risk of earlier onset of menopause than those in the general population (14) and issues related to the menopause can be complicated by methadone treatment (15). Symptoms such as hot flushes resemble symptoms of opiate or methadone withdrawal (16, 17). Women with drug using histories experiencing increased levels of physical discomfort, insomnia, irritability, anxiety, and depression during their menopausal transition may be at higher risk of relapse (15). The social marginalisation of women who use drugs combined with the intersection of being an older woman risks inhibiting wider medical and sociological interest in this particular group of women.

As the site of biographical experiences, bodies are shaped by people's socio-economic and cultural circumstances (18). Midlife can be for some people a critical time to manage both chronic conditions and mental health conditions (19). For people who have used drugs over a long period of time, midlife and older can be a time when the somatic nature of the body becomes more evident. The substance use recovery literature is large and increasing but there are relatively few studies that examine the bodily aspects of recovery. Seminal recovery studies discuss people's health in relation to the impact of drug use and methadone treatment but not their health in recovery (20, 21). While there has been some research that has explored bodies in recovery, this work remains limited. Nettleton et al. (22) found people in recovery endure more bodily discomfort and pain over a longer period compared to people who used drugs. Neale et al. (23) explored how the use of opiates and opioids can mask bodily pains but in recovery, the emergence of aches and pains are perceived as the result of general wear and tear from prior substance use, stress, tension and ageing (23). In order to more fully understand the impact of health on recovery “a fuller appreciation of the lived experiences of recovery must incorporate and give greater attention to the body” [(22), p. 353]. This paper provides that attention by seeking to understand what it means to move from a substance using body to a recovered body as women age into their recoveries from substance use.

Leder's (24) concept of dys-appearing bodies is a useful tool that can provide a phenomenological understanding of women's embodied experiences of drug use and recovery through the temporal phase of mid-life. Leder's theory looks at the human body as a lived structure that is central to our lived experience. The lived body is an embodied self that “lives and breathes, perceives and acts, speaks and reasons” [(24), p. 6]. Bodily dys-appearance occurs “when the body undergoes some disturbance, when it “seizes” our awareness” [(24), p. 70]. “Dys” originates from the Greek prefix, meaning bad or ill. For example, we notice pain and bodily discomfort when they stand out from all the other sensations we experience. They hurt and re-focus our attention inward; re-organising our relationships with lived space and time, others and ourselves. But, pain, discomfort and other bodily sensations can also be difficult to articulate to others (and sometimes ourselves) (25). They can disrupt our connection with the external world, inducing self-reflection and isolation. They constrict our being-in-the-world back to an awareness of our own body and the particular body area where sensations are felt. The spatial world stops being the “centre of purposeful action” [(24), p. 75] and the temporal world contracts. Physical discomfort demands our attention and brings us back to the here and now. Bodily dys-appearance can also be characterised by affective disturbance [(24), p. 85]. Anxiety, for example, manifests itself somatically through a number of bodily symptoms including increased heart rate, feeling nauseous and difficulty breathing. Activities, therefore, are directed towards the goal of removing pain and bodily discomfort. Both health and illness exhibit alienations from the body—in health the body disappears and attention is directed towards the world; in illness and discomfort the body dys-appears and attention is directed inward to the self. So too with affective disturbance—in health, capacity to direct attention towards the world is expanded; in anxiety, depression or other forms of affective disturbance the mind dys-appears and attention is directed inward towards the self.

The lived experiences of pain and bodily discomfort in older women who use drugs is an important but barely explored area of research within the drug research literature. The interactions of older age, accompanying illness, drug use, and recovery are yet to be explored. We know there is some evidence that older drug users are reluctant to ask for help due to perceived or enacted discrimination (26) therefore, as populations age, it is increasingly pressing that the role of somatic dys-appearance during drug use and recovery should be considered by researchers within the spheres of women's health and addiction studies.

## Methods

### Recruitment

Women over age 35 with a history of drug use were recruited to this study. The cut-off age of 35 years could be considered young but the health effects of prolonged drug use and its effect on the ageing body suggests that women who were early onset users and engaged in long-term drug use may have a biological age that is older than their chronological age (27, 28). Long-term drug use is defined as 11 years or more (29). Convenience sampling was utilised in this study, as older people who use drugs and particularly older women who use drugs, are a hard-to-reach group (30). Participation was voluntary and the study was advertised through email distribution by contacts and colleagues; on flyers and posters in locations where people in recovery meet, such as recovery cafes; and posted on online recovery sites. Women who chose to participate were included if they met the following criteria: 1) women with a history of illicit drug use; 2) self-identified as in recovery from drug use (abstinent or low risk use) and 3) were 35 years old or older. Participants were excluded if: 1) they said they did not meet the inclusion criteria; 2) identified as having mental ill health or other issues that might trigger distress during the interview or 3) were non-English speakers. In the event, two women were excluded as neither had engaged in illicit drug use. Once participants agreed to interview, arrangements were made for interviews to be conducted at a venue chosen by the women. Ten women chose their home as the location for the interview; five were interviewed on their work premises; and, four chose to come to the first author's university office. Each interview was carried out in a private space. The interviews were conducted one-to-one by the first author (who has extensive experience of carrying out sensitive interviews) and lasted on average 60 min.

### Topic Guide

The topic guide for the study was piloted and developed through work carried out in a previous study (31). The advantages of piloting the topic guide for this study were that it helped hone the questions, identify challenges in the interview process, and follow up areas of further interest. For example, following the pilot study, more opportunities were created for the women to discuss their experiences of health and therapeutic relationships through their periods of drug use and recovery. To help women feel comfortable to talk about their bodies in recovery, questions were adapted from previous studies with older women to help prompt them to discuss appearance and the corporeal aspects of ageing (32, 33). The opening health question moved from the piloted question “*Can you tell me about any changes to your health as you've stopped using?”* to the more open “*How is your health today? How does it compare to when you were in your twenties?”* Indirectly asking about health promoted discussion around the ageing body without any perception of participant discomfort or reticence.

### Analysis

An inductive and deductive analytical approach was used. An inductive analysis develops concepts and themes from the raw data and is an iterative process whereby the data are collected and analysed simultaneously (34). I Nevertheless, it is also recognised that it is impossible to approach the data without any preconceived ideas of what themes or concepts might emerge from the raw data [(35), p. 210] hence a deductive approach is acknowledged.

The interviews were transcribed verbatim by the first author. The first author checked a sample of five transcripts against the recorded data for quality control to ensure their accuracy. In addition, 17 participants were given the opportunity to read through their transcripts. Two participants did not have an opportunity to check their transcript as there was no way to contact them without going through a third person. Maintaining their confidentiality was crucial and as such their transcripts were not forwarded. Of the 17 who were given the opportunity to read and check their transcripts, 12 took up the offer and five responded positively. No participants raised any issues, corrections or complaints.

The transcripts were analysed using Braun and Clarke's (36) thematic analysis technique. Thematic analysis is a flexible and useful heuristic device for managing and producing a detailed account of data and is widely used within qualitative drug-based research (37–39). The advantage of thematic analysis is its relatively simple yet robust analytical strategy. The interview transcripts were coded thematically through six phases by the first author: familiarisation with the data, transcription, initial coding, searching for themes, reviewing themes, defining themes, and report writing. Transcribed interviews were read through at least twice for familiarisation. At this stage, ideas about themes began to emerge and coding began to take shape. A coding framework was devised to identify themes and sub-themes in the interview transcripts. As this study followed on from a pilot study, a number of top-level themes were already identified from the pilot work and related to the study aims. For example, *a priori* themes included health, ageing, and recovery. These top-level themes were relevant and coded to all participants as they were the main topics discussed in the interviews. The coding was further refined as interviews progressed and with repeated reading of the transcripts. For example, in further iterations the code “*health”* was further refined into additional secondary codes, such as women's experiences of the menopause or drawing on the literature to include the concept of “*dysappearing bodies*.” The first author (AS) maintained a coding book, in which codes, description and examples were detailed and discussed with the co-authors of this paper (GR and LP). The qualitative software package NVivo 11 was used to code and categorise the interview data.

### Ethical Considerations

The study was approved by the University of Glasgow's College of Social Sciences Research Ethics Committee (ApplicationNo:400170200). Women who expressed an interest in the study were given the participant information sheet and given at least two days to read through it and ask questions before signing the consent form. The women were assured complete anonymity, with all identifiable information removed from transcripts, and published materials. All names used are pseudonyms.

## Results

### Sample

Nineteen women with a history of using illicit drugs and recovery participated in the study. The women were aged between 36 and 60 years (mean average age 47). The women resided in a mix of urban (*N* = 10), rural (*N* = 6) and coastal locations (*N* = 3) across the North of England and Scotland. Fifteen women were early onset users, starting drug use in their teens and early twenties. Four women were late onset users, starting in their late twenties and early thirties. The participants used a range of drugs including heroin, powder cocaine, crack-cocaine and skunk weed between 7 and 47 years (mean average 21 years). The mean average age at which the women gave up drug use was 34 years old, ranging between 26 years and 54 years of age. Time in recovery ranged from 6 months to 18 years (mean average 9 years). Sixteen women reported no drug use and three women reported occasional low-risk drug use including intermittent use of cocaine, cannabis and alcohol, amphetamines and heroin (low-risk defined as illegal drug use at minimum level causing no psychological, legal, employment, family or health problems [(40), p. 83]).

### Health and Bodies in Recovery—Temporality and Power

The women in this study reported a range of mental health conditions including anxiety, bi-polar personality, schizophrenia, and depression that many had experienced prior to recovery, and some prior to their onset of drug use. Most had experienced or witnessed physical and/or sexual violence as children and/or adults. At least seven women had been prescribed anti-depressants for decades. Nonetheless, most of the women described improvements in mental health after they stopped using drugs although this occurred gradually over time in recovery. Discussing early recovery and mental health well-being some of the women recalled their vulnerability in relation to intimate relationships.

“*I would say the first couple of years in recovery. It's a very dangerous place, psychologically.”* (Fiona, age 44, recovery 17 years)

“*I had a period of time a few years ago when I was completely clean and some of the behaviours, some of the stuff that I was doing with men, using men to make me feel good...I suppose it was a bit like, it was addictive behaviour you know…I think I had some kind of breakdown or something and obviously I relapsed...”* (Claire, age 39, recovery 18 months)

Viewing from the present her behaviours in previous recovery as “*dangerous*” and “*crazy*,” Claire actioned intimacy with men to improve her self-esteem and sense of self-worth. For Jennifer, aged 44, coming to terms with her past meant coming to terms with her body:

“*I think that those behaviours that I learned to manage life for a long time, see my body as a sexual object, something that I used in some transaction, those behaviours were probably the hardest ones to change. Stopping drugs was hard but even when I stopped drugs I continued those behaviours at periods you know here and there. So I think today I see my body as, it's just a body. It doesnae have any power or anything like that...It took me a long time to connect with my body and feel like I was alright with it.”* (Jennifer, age 44, recovery 10½ years).

Learning how to manage emotions and subsequent behaviours in ways that did not leave them feeling exploited (either by their own actions or by those of others), was important in enabling some of the women to come to terms with their bodies in the past and appreciate them in the present: thus, improving their sense of bodily self-efficacy and self-esteem.

In terms of physical health, some of the reported conditions included chronic obstructive pulmonary disease (COPD) and emphysema which some participants attributed to smoking drugs; nerve pain and deep vein thrombosis occurring from injecting drug use; and trigeminal neuralgia and fibromyalgia, rheumatoid arthritis and arthritis that occurred, or the women became aware of, in recovery. Seven women had cleared the hepatitis C virus (HCV), contracted during their period of using drugs. No women had hepatitis B or C at time of interview. While most of the women described their current physical health as good there were concerns that irreparable damage had been done to their bodies as a consequence of their drug use. Women in both long-term and early recovery expressed concern that risk behaviours in the past might have consequences for their health in the future:

“*It does concern me that ehm…there's maybe some damage that I've done maybe in my past or ehm that's going to come back and bite me on the arse. That's going to come back ehm and kill me maybe...I'm constantly aware of my mortality now I think. And I think that's because I'm fucking over 50. And I just think about how careless I was with my health and my life.”* (Lorna, age 53, recovery 18 years)“*I've got a lot of fear as well you know because my family all die young with cancer you know…and I went for genetic testing and they said I've got a 80% chance of lung cancer but didn't stop me using or smoking or anything like that. Now I'm more thinking about it now. I'm like that “oh fuck” you know. But what's for you is for you, you know what I mean. That's the way I've got to think. I cannae sit and just dwell about it.”* (Sara, age 44, recovery 18 months)

Both Lorna and Sara felt a sense of their own mortality as women in mid-life: Lorna because she was “*over 50*” and Sara as she approached her mid-40s. Lorna's “*concern*” that past “*careless*” behaviours had contributed to known (HCV) and unknown health conditions was underpinned by the helplessness she felt. Sara's fear was compounded by a family history of cancer and while worried about the consequences of her cocaine use, knowing there could be a genetic propensity to cancer, she accepted it as something she had no control over. Both women felt a sense of powerlessness regarding their future health status. Nonetheless, Sara and Lorna's narratives illustrate how some women make sense of their health in the present, which is anchored to behaviours in the past that have potential to impact on health and well-being in the future. Moreover, the pervasive social value of individuals taking personal responsibility for their health may be such that, no matter how long women have been in recovery, former actions, lifestyles and behaviours that were potentially physically injurious to health and well-being are used to explain current health conditions by the women themselves and their health providers (41).

Perhaps because of a tendency to accept their current health as a consequence of past behaviours, some of the women compared their health to other people who use drugs and spoke about pain and illness as something to be endured. A number of the women described their health as good although they suffered pain on a daily basis.

“*Aye I've got a sore hip but on the whole I'm not too bad. I have got chronic lower back pain...I've got asthma, but on the whole my health's really good. And I know I'm lucky and I'm grateful because I know a lot of people at my age whose health is not nowhere near as good and you lose a lot of people. There's always someone passing away. Like every week there's someone else.”* (Grace, age 49, recovery 5 years)

“*But like I says my health now, obviously I'm feeling all the aches and pains and whatnot…All the damage I've done to my nerves with all the injecting in my legs and things like that. Ehm but I'm not really complaining either. Especially when I think back what I put my body through and how I've come out the other end of it....”* (Terri, age 59, recovery 10 years)

Some of the women were stoical regarding their current health, “*grateful”* for having survived their drug use relatively unscathed. It has been theorised that certain types of pain and illness (such as that associated with substance use) are experienced as ‘moral events' involving internalised and externalised shaming and blaming (42). A tendency to judge people who use drugs, plus the stigma associated with injecting drug use and bloodborne viruses is such that some people may be reluctant to discuss health issues anticipating some form of felt or enacted stigma from others (43). Furthermore, emphasising personal responsibility for their health, risks disregarding the political, economic and socio-cultural circumstances of people's lives (44). Maya, age 42, recommended that practitioners working with older women who use drugs should remember, “*We are people with complex issues and often very challenging histories, not necessarily of our own making.”*

### Ageing in Recovery

Some women spoke about changing their outlook on their health as they got older. Acknowledging potential damage to their health from past drug use, the women now took steps to improve their health in the present.

“*I feel like my attitude towards my health, I would never, like I went for a smear test last Tuesday. I never miss anything. I feel really privileged to be in a country where we get those regular checks for free. Ehm, so I buy into all of them.”* (Jennifer, age 44, recovery 10½ years)

“*As I've got older my health has become more important...I think gradually there's been an awakening of self-worth that's directly related to self-care.”* (Janine, age 47, recovery 21 years)

Janine's attention to her health was prompted by her sponsor in Alcoholics Anonymous who encouraged good nutrition, exercise and sleep. From Janine's perspective, changes to her diet and general self-care had led to improvements in both her health and her sense of deserving care: “*I was worthy of that care.”*

Abstaining from drugs and entering recovery revealed aches, pains and other symptoms that were absent while the women were using drugs.

“*The first thing that I noticed when I got clean was that life hurts....I'd been using opiates for years and years and years. Not feeling any pain apart from the pain of withdrawal. And once that's over I realised, my god, my bones have aged somewhat…I felt like an old woman complaining to the doctor “This is sore and that's sore.”*” (Lorna, age 53, recovery 18 years)

“*I never thought I had bad health until I stopped...Soon as I stopped oh my god. I think I went from a 20 year old to an 80 year old. Just the pains you get and you're beginning to realise what other things you have, things that are wrong with you that you didn't think you had.”* (Shona, age 60, recovery 12 years)“*I think when I was using drugs, it masks so much doesn't it”* (Kate, age 60, recovery 5½ years)

The women likened their painful bodies to old bodies and recovering bodies. Lorna, who stopped using illicit drugs at age 35 and Shona, who stopped at age 48 both described their drug-free bodies (in early recovery) as “old.” Applying Leder's (24) hypothesis, their ageing, recovering bodies seized the women's attention. During their drug using period, their bodies went unnoticed until withdrawal pains seized their attention. In recovery, with no opiates or other self-medicating drugs in their system, pain or discomfort were noticed when they interrupted their consciousness. At this point the women's bodies were no longer “absent,” instead they disturbed or “dys-appeared” (24).

In addition to a reduction in or absence of illicit drug use, the decrease or absence of prescribed drugs also heightened the women's awareness of pain.

“*Back then ehm I…when I was obviously getting so much pain killers, taking my painkillers and Valium, never felt pain eh. Never felt anything eh. Total numb feeling it was, I suppose. But now I'm in pain all the time.”* (Shona, age 60, recovery 12 years) “*And that's the other thing because I am older and I'm weaning myself off this pain medication, when I was young I never had any aches and pains. I was like ‘oh fuck' you know so there is this as well. I think your body gets used to aches and pains but basically because I've anaesthetised myself for so many years, I'm just, can't cope with it. So that does concern me a bit.”* (Nina, age 55, recovery 12½ years)

Nevertheless, some of the women were reticent to blame all their aches and pains on the absence of drugs and felt that they were experiencing the “natural” bodily discomforts of the ageing process. As Shona stated, “*I put a lot of things down to I'm getting old because I am.”* (Shona, age 60, recovery 12 years).

### Ageing Into the (Peri-) Menopause

The women in this study were at different stages in their reproductive lifespan. Some were still menstruating or entering into the peri-menopause while a few were post-menopausal. This section reveals some of the women's discussions around menstruation and the menopause and its impact on their recovery.

Women who use drugs can experience amenorrhoea, the absence or cessation of menses (periods) in women of reproductive age (12). For Jennifer (age 44, recovery 10½ years), recalling her absence and thinking about her embodied future, this meant:

“*All that weight loss to not having periods for years. That's osteoporosis in the post.”* (Jennifer,

Perceived infertility due to drug-induced amenorrhoea may result in unplanned pregnancy (12) during active drug use and following abstinence when the women's menstrual cycle begins again. Fiona found out she was 26 weeks pregnant following detoxification from methadone, heroin and diazepam and said:

“*I didn't realise I was actually pregnant because I'd not had regular periods for about 2 years.”* (Fiona, age 44, recovery 17 years)

The return of women's periods after years of drug use can be an uncomfortable aspect of recovery, as described by Claire:

“*PMT is horrendous. I dread it. And it seems to surprise us each month. I'm like what's going on, why do I feel like this and why is my head racing...So yeah, I really struggle with it. It's, you know, this recovery stuff, getting used to feelings and thoughts anyway, it's hard enough without all that going on. PMT stuff...Yeah. I don't find it easy this time of the month I really don't. And I think that anxiety gets a lot more when I'm due my period.”* (Claire, age 39, recovery 18 months)

These findings support other studies on women recovering from heroin use (23). For some women in early recovery who are learning to manage their emotional well-being, menstruation involves a process of recognising, remembering and self-discipline. Insights into the potential for relapse caused by the return of menstrual symptoms and the anxiety that can accompany them suggest this is an area of women's recovering health that requires further enquiry.

Seven women discussed their experiences of the peri-menopause, the transition time to the menopause, when menstruation ceases altogether. Jennifer used a combination of hormone replacement therapy (HRT) and mindfulness practise to manage her symptoms which she described as “*extreme*” and having “*a significant impact on* [her] *well-being*.” Symptoms included brain-fog, anxiety, fatigue and loss of libido.

“*My GP initially diagnosed me as being depressed and I was offended almost because I knew I was feeling a bit down but I didnae feel like I was depressed. This was a male doctor and I sought a second opinion and went to a female doctor and she got it instantly and she went “no, you're not depressed this is what's going on for you.” And just to have someone to validate that and go “yep, this is.” And I thought that's what it was.”* (Jennifer, age 44, recovery 10½ years)

While Jennifer attributed her low mood to the changes that come with (peri-) menopause, her experience echoes those of women elsewhere with regard to health concerns diagnosed as psychogenic by medical practitioners (16, 45). Jennifer felt that the menopause was an issue that needed to be discussed with women in recovery:

“*I think that menopause as well is something that should be talked about...especially for women in recovery. I think you're programmed to always bring things back to yourself and look at yourself and what's going on with you then going through this period of my life this is not something that is the result of my past or, it's just something that all women go through.”* (Jennifer, age 44, recovery 10½ years)

The menopause, as Jennifer points out, is a natural bodily transition that requires medical and/or social support and understanding in the present, not personal self-reflection on past or present behaviours or actions. Like Claire, around the start of her periods, who experienced sensations that felt like the more familiar sensations of withdrawal and cravings, Grace recalled her menopause symptoms as similar to being “*on something”* and found it worrying and uncomfortable.

“*I just felt like I had baby brain. Everything was getting on top of me. I was getting forgetful. I was thinking I was on something when I wasn't. Just I don't know. Couldn't concentrate. Wasn't sleeping properly. Hot flushes. Over and over. They just got worse and worse and worse. Headaches. Restless. Emotional.”* (Grace, age 49, recovery 5 years)

These findings show that older women in recovery from drug use can experience menstrual, peri-menopausal and menopausal symptoms that feel, to them, similar to the effects of narcotic drugs and drug withdrawal. This presents not only a potential relapse trigger, but also a set of symptoms that are open to misinterpretation by a medical profession that already has a history of dismissing women's understandings of their own bodies as psychogenetic (45), and specifically of dismissing the sense-making of women in recovery through an addiction and recovery lens (46). Together, these produce a double barrier to care, as women and care providers both make sense of menstrual, peri- and menopausal symptoms through both a substance use and wider psychogenetic lens.

Having considered the women's self-reported health and experiences of their bodies and ageing in recovery, the next section explores trauma as felt and embodied by the women.

### Embodied Trauma, Embodied Emotions

Most of the women discussed having experienced some form of physical or psychological trauma. In the *Body Keeps the Score*, Van der Kolk (47) suggests post-traumatic stress disorder (PTSD) is embodied and carried on in the body long after the trauma has stopped. Terri, a child-rape victim expressed how that event had affected her health and that even with counselling the physical and mental health effects of the trauma remained:

“*It's the amount of years that it's took that's, it's a hard part as well. And it's all the drugs and the drink and how it affected my health as well into the bargain. Because I'm in pain every day, every day. Seven days a week...And it's just so, so unbelievable how much that could just change your life like that. Ken, somebody could sit and speak to you for hours but when that person goes away it's just there again.”* (Terri, age 59, recovery 10 years)

Terri was particularly graphic in describing the intense embodied emotions she still experienced in times of distress. A few months earlier, she had been abused on social media.

“*Oh I was devastated....And I was like I was drained with it…totally drained. I was really gutted about the whole situation like. And it went from there to they were actually going to put me under the counsellor because I told the doctor “that is on my mind in the morning when I wake up. When I go to my bed.” I said “I am so embarrassed that I feel humiliated at all the amount of people that's joined in and that. Honestly” I says “I feel absolutely gutted wi this.” And I actually lost weight just in that wee space of time wi the stress of it. I'll never forget it. I'll no, that'll never just go away.”* (Terri, age 59, recovery 10 years)

The embodied emotions Terri felt had a temporal aspect that was not easily shaken. Terri imagined she would never forget how she felt, her embodied humiliation carried into the future. Time contracts as embodied emotions and trauma are felt and relived. The past, present and future seemingly combine. Maya described how images evoke painful memories for her:

“*Like I was saying with the photo…because how horrible the image it creates for me in my mind you know I can see myself, I can remember the feelings and how I felt at that time and it was so horrendous.”* (Maya, age 42, recovery 14 years)

Despite all the work the women have put into their recovery, the trauma they have experienced throughout their lives, is still carried within their bodies. Research elsewhere has described the return of emotions in early recovery as something akin to becoming “un-numb” [(23), p. 93]. Eventually, emotions settle and people learn how to manage them more effectively. The findings in this study echo those of Neale et al. (23) but also demonstrate that even in long-term recovery the body still “keeps the score” (47) when remembering past events and traumas.

## Discussion

The interviews illustrate the complex bodily processes felt by women in recovery from substance use. The women described a number of mental and physical health symptoms that have also been identified in other studies with substance users (4–6, 48). However, this study moves beyond describing health conditions to explore mid-life and older women's bodily experiences of drug-free and ageing bodies. The long-term effects of prolonged illicit and prescription drug use are concerns for these women, as is the cessation or reduction of some prescribed medications. They work hard to make sense of bodily sensations [around pain, discomfort, menstruation and the (peri-) menopause] and navigate these in relations with others—including medical practitioners who frame them as psychogenetic and/or related to their past drug use. The intersection of the ageing and drug-free body brings to the foreground changes in the women's values towards their health where greater self-care is taken, and a process of self-understanding is undertaken. As younger women who used drugs, the body was, as Leder describes it, absent (24). Nevertheless, while their bodies went unnoticed in terms of pain and illness, their bodies were still valued in terms of transactional power. As older drug-free women, improvements in self-worth combined with a sense of personal responsibility for their health, helped them re-evaluate their bodies. For some women, this instilled feelings of greater bodily and emotional control that were absent through their drug-using period. However, ideas about personal responsibility were complicated. They were also at times related to an awareness of the wider social economic and political circumstances of the women's lives. In this sense, emphasis on individual responsibility risked downplaying factors over which the women had no control, and also heightened the risk for feelings of stigma and shame. Understanding of these wider factors that generated health risks was also important for developing ideas about being worthy of care. Some actively worked out to maintain good physical health and almost all practised some form of meditative or therapeutic practise to maintain good mental health. Despite their new and emerging pains, their bodies in recovery were preferable to their bodies in active drug use. This re-evaluation of bodies that are free from unmanageable and damaging drug use could provide opportunities for improving medical practise and recovery support with mid-life and older women. Understanding women's medical and drug-using histories is obviously important but as highlighted in the findings, can also be reductive. Practitioners could build on women's emerging and growing sense of worth and motivation for improving self-care by acknowledging and paying attention to the women's own understanding of their bodies and health. Advice and support around the bodily changes that abstinence incurs could help women make sense of these changes, reducing perhaps some of the discomfort and anxiety they experience. More attention could be given to diet, physical activity and good mental health practises (such as mindfulness) which might help women manage more effectively the emerging and often uncomfortable, bodily sensations of early recovery.

Paying attention to the women's voices we hear how the body and health in recovery involves much more than abstinence from drugs. Moving from long-term substance use and into recovery is potentially a time of increased vulnerability for some women as their libido returns and they reach out for intimacy and support [(49), p. 23]. Understanding the body and their sexuality as it was in the past and its centrality to feelings of self-worth and self-esteem required the women in this study to learn how to deal with their emotions and bodily sensations without feeling bodily exploited. To what extent women struggle with this aspect of early recovery is relatively unknown but the findings from this study suggest that some women will find it challenging. Research into this aspect of women's early recovery could help raise awareness and inform practise around this period of potential vulnerability helping women avoid repeating negative behaviours from their past (50).

Most women approaching or in mid-life manage changes in their reproductive cycles. Menstruation and menopause are events that can interrupt and disrupt women's bodies and where symptoms need to be re-interpreted as natural bodily processes. The women in this study discussed their menses and (peri-) menopausal symptoms, shedding further light on a neglected aspect of research in the addiction literature. Adding to the work of Tuchman (16) and Johnson et al. (17), the women confirmed the “felt” similarities between the effects of drug use and withdrawal and the symptoms of pre-menstrual tension and the peri-menopause/menopause. They further reveal how these symptoms can be particularly challenging for women in early recovery. They have to learn to understand and interpret new and emerging bodily pain and sensations without the anaesthetising effects of drugs. Understanding these as natural could help reduce anxiety for women in active drug use, medication-assisted treatment, and recovery. Research that explores women's experiences of their reproductive lifecycles could help to reveal the complex intersections of gender, health and ageing in drug use and recovery. Insights gathered from women who use or have used drugs, as well as professionals in the fields of substance and use and/or women's health, could support the development and co-production of information materials, awareness-raising, and possible interventions and/or support around menses, the perimenopause and menopause. Implications for practise suggest treatment staff and prescribers are trained to listen to and support women, and transfer up-to-date knowledge on this aspect of women's health.

Moving from the body in active drug use to the body in recovery is not without its challenges for mid-life and older women, and for the practitioners who support them. New sensations and feelings, physical and mental, must be re-interpreted in light of their ageing and drug-free bodies. It is the body that carries them through their experiences and it is the body through which they experience the journey. Their bodies contain their past, present and future selves. It symbolises who they were, who they are and who they might become. Understanding what it feels like for women making this transition from substance use to recovery is relatively uncharted in the literature to date. Women's embodied recovery is about far more than just abstinence. As Maya reminds us, practitioners need to acknowledge that women, who have used or use drugs, often have challenging and complex histories unrelated to their drug use. Practitioners must pay attention to women's own understanding of their bodies, bodily sensations and physical health needs in order to provide them with effective health care and support as they move out of drug use, into and through recovery.

### Limitations

The sample size of 19 women might be considered a limitation of the study however, the original sample size (15 to 25 women) was chosen to reflect the anticipated challenges of recruiting older women with a history of drug use (30). A larger sample of women from across the UK and a greater number of women in active drug use would provide a wider range of experiences to explore. Speaking with a greater number of women in their fifties and sixties could also elicit further information on the peri-menopause and menopausal period in women's lives, thus adding to the limited number of existing studies, most of which originate from the USA. Moreover, including older women from other marginalised groups such as homeless women, BAME women and women in the criminal justice system could add insights into the structural and socio-economic barriers and inequalities that impact on bodily recovery among women with a history of drug use. As such, the findings of this study, as with qualitative inquiry in general, cannot be said to represent the universal experiences of all older women with a history of drug use, although the methods used to collect the data and the findings could be transferable to health-based studies with populations of older women who use drugs elsewhere in the UK and beyond. Nevertheless, despite the size of the sample, the participants in this study reported having to learn how to understand their recovered or recovering body, how to interpret and respond to often novel and/or unwelcome bodily sensations, and to do so in relation to health care providers and others.

### Final Thoughts

This paper explores from women's standpoints and within a context of ageing and health, journeys from being a woman who uses drugs to a woman in recovery, providing greater depth and understanding on, an issue that is relatively unknown in UK substance use research. Paying attention to the women's voices provides unique perspectives on their embodied recoveries, as felt and experienced by them. The women's experiences are singularly personal to them, yet taken together they offer deeper understanding of the bodily experiences that shape their health as they transition into and through recovery. In doing so, the paper provides an original and important contribution to studies that explore the intersection of gender, substance use, ageing, recovery and the body.

## Data Availability Statement

The data that support the findings will be available in University of Glasgow's repository for research data (Enlighten: Research Data) and the UK Data Service (University of Essex) following a 24 month embargo from the date of publication to allow for publication of research findings. Requests to access the datasets should be directed to ashaw419@gmail.com.

## Ethics Statement

The studies involving human participants were reviewed and approved by University of Glasgow's College of Social Sciences Research Ethics Committee. The patients/participants provided their written informed consent to participate in this study.

## Author Contributions

AS is responsible for the conceptualisation, design of the study, fieldwork, data management, analysis, and drafted the first version. LP and GR critically revised and finalised it for publication. All authors approved and contributed to the final version.

## Funding

This Ph.D. study was funded by the Economic and Social Research Council [ESRC (DTC) Main ES/J500136/1].

## Conflict of Interest

The authors declare that the research was conducted in the absence of any commercial or financial relationships that could be construed as a potential conflict of interest.

## Publisher's Note

All claims expressed in this article are solely those of the authors and do not necessarily represent those of their affiliated organizations, or those of the publisher, the editors and the reviewers. Any product that may be evaluated in this article, or claim that may be made by its manufacturer, is not guaranteed or endorsed by the publisher.
